# Multi-Omics Analysis of Molecular Characteristics and Carcinogenic Effect of NFE2L3 in Pan-Cancer

**DOI:** 10.3389/fgene.2022.916973

**Published:** 2022-06-29

**Authors:** Mengxiao Liu, Hui Wei, Jing Yang, Xia Chen, Haoying Wang, Ya Zheng, Yuping Wang, Yongning Zhou

**Affiliations:** ^1^ The First Clinical Medical College, Lanzhou University, Lanzhou, China; ^2^ Department of Gastroenterology, The First Hospital of Lanzhou University, Lanzhou, China; ^3^ Key Laboratory for Gastrointestinal Diseases of Gansu Province, The First Hospital of Lanzhou University, Lanzhou, China

**Keywords:** NFE2L3, pan-cancer, multi-comics, tumor immunity, DNA methylation

## Abstract

NFE2L3, also known as NFE2L3, is a nuclear transcription factor associated with the pathogenesis and progression of human tumors. To systematically and comprehensively investigate the role of NFE2L3 in tumors, a pan-cancer analysis was performed using multi-omics data, including gene expression analysis, diagnostic and prognostic analysis, epigenetic methylation analysis, gene alteration analysis, immune feature analysis, functional enrichment analysis, and tumor cell functional status analysis. Furthermore, the molecular mechanism of NFE2L3 in liver hepatocellular carcinoma (LIHC) was explored. The relationship between NFE2L3 expression and survival prognosis of patients with LIHC was analyzed and a nomogram prediction model was constructed. Our study showed that NFE2L3 expression was upregulated in most cancers, suggesting that NFE2L3 may play an important role in promoting cancer progression. NFE2L3 expression is closely related to DNA methylation, genetic alteration, immune signature, and tumor cell functional status in pan-cancers. Furthermore, NFE2L3 was demonstrated to be an independent risk factor for LIHC, and the nomogram model based on NFE2L3 expression had good prediction efficiency for the overall survival of patients with LIHC. In summary, our study indicated that NFE2L3 may be an important molecular biomarker for the diagnosis and prognosis of pan-cancer. NFE2L3 is expected to be a potential molecular target for the treatment of tumors.

## Introduction

Globally, cancer is one of the most severe diseases threatening human health and poses a heavy health and economic burden to society as a result of its high morbidity and mortality (Sung et al., 2021). Most malignant tumors are in the intermediate and advanced stages when clinically diagnosed, which is often too late to treat the patients effectively. Although significant progress has been made in cancer therapy in recent years, the prognosis of most cancer patients remains poor due to several contributing factors, including drug resistance, side effects of drugs, and chemosensitivity (Liu et al., 2020a). Therefore, it is necessary to develop new biomarkers and therapeutic targets for early cancer screening and treatment.

NFE2L3 is a member of the Cap“n”Collar (CNC) family, which belongs to the basic region-leucine zipper (bZIP) transcription factor (Chevillard and Blank, 2011). The NFE2L3 gene is located on the human chromosomes 7p15-p14 and encodes a 694-amino-acid protein (Kobayashi et al., 1999). Glycosylation is an important modification form of NFE2L3 (Nouhi et al., 2007). Previous studies have demonstrated that NFE2L3 plays an important role in inflammation and is identified as a stemness marker gene as it is upregulated in the early stage of stem cell differentiation (Witschi et al., 1989; Braun et al., 2002; Byrne et al., 2007; Ben-Yehudah et al., 2010). Studies have also shown that NFE2L3 is closely related to the development and progression of tumors (Saliba et al., 2022). Knockdown of NFE2L3 showed a tumor-suppressive effect in liver and gastric cancer cells (Yu et al., 2019; Wang et al., 2021). NFE2L3 has also been identified as a novel DNA methylation driver gene and prognostic marker of kidney renal clear cell carcinoma (KIRC) (Wang et al., 2019). Another study showed that the β-catenin/Tcf4 complex promoted the proliferation of colon cancer cells and upregulated GLUT1 expression by upregulating the expression of NFE2L3 mRNA (Aono et al., 2019). However, there are limited studies on NFE2L3 in different cancer types, thus it is necessary to comprehensively analyze NFE2L3 expression in pan-cancers. This will aid in exploring effective prognostic biomarkers and provide future reference data for further investigations.

Our study comprehensively analyzed NFE2L3 expression in pan-cancers using different databases. The diagnostic and prognostic value of NFE2L3 in pan-cancers was also evaluated. The correlations between NFE2L3 expression and DNA methylation, genetic alterations, immune features, and tumor cell functional status in pan-cancers was also investigated. A gene function enrichment analysis of NFE2L3 in pan-cancer was performed and the molecular mechanism of NFE2L3 in LIHC was evaluated. NFE2L3 was identified as an independent risk factor for OS in LIHC. In conclusion, the study revealed the essential biological function of NFE2L3 in pan-cancers and showed that NFE2L3 has the potential to be used as an effective therapeutic target and tumor diagnostic marker.

## Materials and Methods

### Gene Expression Analysis in Pan-Cancer

Differential expression of NFE2L3 in normal human tissues was analyzed using the Human Protein Atlas (HPA, v21.0) database (https://www.proteinatlas.org/) (Thul et al., 2017). RNA-seq data and relevant clinical information related to the differential expression of NFE2L3 in various tumor cell lines were obtained from the Cancer Cell Line Encyclopedia (CCLE) database (https://sites.broadinstitute.org/ccle) (Barretina et al., 2012). The differential expression of NFE2L3 between tumor and normal tissues across The Cancer Genome Atlas (TCGA) pan-cancer was analyzed using the TIMER2.0 database (http://timer.comp-genomics.org/) (Li et al., 2020). Analytical data for differential expression of NFE2L3 in paired pan-cancer samples were accessed from TCGA data portal (https://www.cancer.gov/). The subcellular localization of NFE2L3 was investigated using the HPA database and the differential expression of NFE2L3 protein in various tumor and normal tissues was analyzed. R software v3.6.3 and the ggplot2 package v3.3.3 were used for statistical analysis and visualization. P-values were calculated with the Wilcoxon signed rank test and Wilcoxon rank sum test, and *p* < 0.05 was accepted as statistically significant (ns, *p* ≥ 0.05; ∗*p* < 0.05; ∗∗*p* < 0.01; ∗∗∗*p* < 0.001; ∗∗∗∗*p* < 0.0001) (Stanley et al., 2020).

### Clinicopathological Correlation and Survival Prognostic Analysis

The RNA-seq data (level3) and related clinical information were obtained from TCGA cohort. The relationship between NFE2L3 expression and survival prognosis in patients with pan-cancer was analyzed using univariate Cox regression and the results were visualized using the “forest plot” R package. The differential expression Wilcoxon signed rank analysis of NFE2L3 among tumor pathological stages was performed on Gene Expression Profiling Interactive Analysis v2.0 (GEPIA2) (http://gepia2.cancer-pku.cn/#index) (Tang et al., 2019). P-values < 0.05 were considered statistically significant.

### Diagnostic Value Analysis

Receiving operating characteristic (ROC) curves were constructed to evaluate the diagnostic value of NFE2L3 in multiple tumors. The area under the ROC curve (AUC) was rated as outstanding discrimination (AUC ≥ 0.90), excellent discrimination (0.8 ≤ AUC < 0.9), acceptable discrimination (0.7 ≤ AUC < 0.8), and poor discrimination (AUC < 0.70). The pROC package v1.17.0.1, and the ggplot2 package v3.3.3 were used for analysis and visualization, respectively.

### Epigenetic Methylation Analysis

Methylation data were obtained from the Illumina Infinium DNA methylation platform arrays HumanMethylation450. DNA methylation levels of NFE2L3 in TCGA pan-cancer were analyzed using the SMART database (http://www.bioinfo-zs.com/smartapp/) (Li et al., 2019). Comparison of DNA methylation levels in 19 methylation CpG sites of NFE2L3 in TCGA pan-cancer (PANCAN) cohort was performed using the UCSC Xena database (http://xena.ucsc.edu/) (Goldman et al., 2020). The correlation between DNA methylation levels of NFE2L3 and mRNA expression of NFE2L3, tumor cell dryness, immune subtypes, and survival analyses of differential DNA methylation levels in TCGA pan-cancer (PANCAN) cohort were investigated using UCSC Xena. Moreover, the analysis results were visualized through GraphPad Prism v8.4.2 and the survminer package v0.4.9 (ns, *p* ≥ 0.05; ∗*p* < 0.05; ∗∗*p* < 0.01; ∗∗∗*p* < 0.001; ∗∗∗∗*p* < 0.0001).

### Analysis of Protein Topology Mutants and Genetic Alterations

The Protter database (https://wlab.ethz.ch/protter/start/) is a web-based application tool used for visualizing the sequences, topologies, and annotations of individual proteins (Omasits et al., 2014). The variation in NFE2L3 protein topology was evaluated using the Protter database and a schematic representation of the secondary structure of the NFE2L3 protein was generated. In addition, the genetic alterations of NFE2L3 in pan-cancers were analyzed using cBioPortal (http://www.cbioportal.org/) (Cerami et al., 2012; Gao et al., 2013).

### Immune Feature Analysis

The correlation between NFE2L3 expression and immune cell infiltration, immune checkpoints, immunosuppressive cell infiltration, and immune cell markers in TCGA pan-cancer was analyzed using the TIMER database. The results of the analysis were visualized using heat maps of Spearman’s correlations that were generated from the ggplot2 package v3.3.3. Furthermore, the differential expression of NFE2L3 in different molecular subtypes and immune subtypes in pan-cancer were analyzed using the TISIDB database (http://cis.hku.hk/TISIDB/) (Ru et al., 2019). Based on previous studies and on RNA-seq data from TCGA pan-cancer, the correlation between NFE2L3 expression and tumor mutational burden (TMB) and microsatellite instability (MSI) was analyzed and visualized by R software (version 3.6.3) (Bonneville et al., 2017). The potential of NFE2L3 as a biomarker for predicting responses to tumor immune checkpoint blockade therapy was evaluated using the TIDE database (http://tide.dfci.harvard.edu/). In addition, the correlation between NFE2L3 expression and immune checkpoint blockade (ICB) overall survival outcome was analyzed in the immunotherapy dataset and the ability of NFE2L3 knockdown to regulate lymphocyte-mediated tumor killing efficacy in the CRISPR Screen dataset was evaluated (Jiang et al., 2018). All correlation analyses were performed using Spearman’s correlation test. P-value < 0.05 was considered to be statistically significant.

### Functional Status Analysis of Tumor Cells

The correlation between NFE2L3 expression and functional status of multiple tumor cells based on single-cell sequencing data was analyzed using the CancerSEA database (http://biocc.hrbmu.edu.cn/CancerSEA/) (Yuan et al., 2019).

### Functional Enrichment Analysis

GeneMANIA was used to identify the top 20 genes related to NFE2L3 (Warde-Farley et al., 2010). STRING was used to conduct the protein-protein interaction (PPI) network by setting the following main parameters: minimum required interaction score (“0.300”) and maximum number of interactors (“no more than 50 interactors”). Visualization of the PPI network was constructed using Cytoscape. The top 100 genes with a similar expression pattern to NFE2L3 were surveyed using the GEPIA2 database (Szklarczyk et al., 2019). In addition, the intersection of genes related to NFE2L3 obtained from the three databases were identified with results being visualized with a Venn diagram. The correlation between the expression of NFE2L3 and the intersection of the genes related to NFE2L3 obtained from the above three databases was analyzed using the GEPIA2 database. Gene Ontology (GO) and Kyoto Encyclopedia of Genes (KEGG) pathway enrichment analyses was performed for NFE2L3-related genes. The ggplot2 package v3.3.3 was used for visualization and the cluster Profiler package v3.14.3 was used for statistical analysis. A *p*-value < 0.05 was considered to be statistically significant. In addition, tumor patients in TCGA cohort were divided into high and low expression groups according to the median expression level of NFE2L3 mRNA, including BRCA, CHOL, ESCA, HNSC, KIRC, PRAD, UCEC, and THCA. The differentially expressed genes in the high and low expression groups in the multiple tumors described above were analyzed using the DESeq2 package (version 1.26.0). GSEA analysis of the differentially expressed genes was performed in these tumors using the clusterProfiler package (version 3.14.3).

### Co-Expression Genes and Differentially Expressed Genes Analysis in Liver Hepatocellular Carcinoma

The top five genes positively associated with NFE2L3 expression and the top five negatively associated genes in LIHC were evaluated. The results were visualized using a heat map with Spearman’s correlation. Furthermore, TCGA-LIHC cohort was divided into a high and low expression group according to the expression level of NFE2L3 mRNA, and the differentially expressed genes (DEGs) between the high and low expression group of NFE2L3 in LIHC were analyzed (Love et al., 2014). The results were visualized using a volcano map with the following threshold values: |log2 fold-change (FC)| > 2.0, and adjusted *p*-values < 0.05. GO and KEGG enrichment analyses were performed for the DEGs that met the screening requirements. In addition, all the differential genes between the high and low expression groups of NFE2L3 were included in the GSEA analysis. The top 10 signaling pathways from the analysis were presented as merged plots of GSEA. R (version 3.6.3) was used for statistical analysis and visualization, and the main R package that was used included the ggplot2 package v3.3.3 and the cluster Profiler package v3.14.3.

### Prognostic Model Based on NFE2L3 Expression and Clinical Characteristics in Liver Hepatocellular Carcinoma

The correlation between NFE2L3 expression and multiple clinical characteristics of LIHC were analyzed using logistic regression. The factors associated with the overall survival (OS) of patients with LIHC were analyzed using univariate and multifactorial Cox regression. In addition, a nomogram integrating NFE2L3 expression and the prognostic factors of the multivariable model for OS in LIHC from TCGA data was created using the nomogram function from the RMS package v6.2-0 and survival package v3.2-10. The accuracy of the nomogram model for predicting overall survival of patients with LIHC was evaluated using calibration curves. The related RNA-seq and clinical data were accessed from TCGA database, normalized as transcripts per million (TPM), and then log2 transformed. The Wilcoxon signed rank test was used to determine statistical significance (ns, *p* ≥ 0.05; ∗*p* < 0.05; ∗∗*p* < 0.01; ∗∗∗*p* < 0.001; ∗∗∗∗*p* < 0.0001).

## Results

### The Expression of NFE2L3 in Pan-Cancer

**FIGURE 1:**
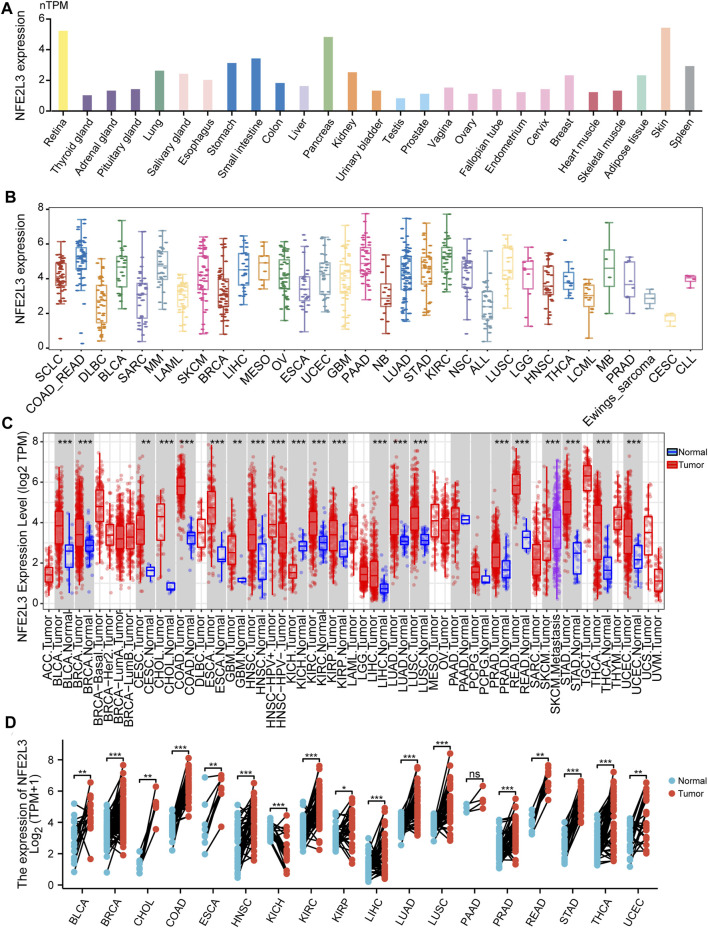
Expression level of NFE2L3 in pan-cancer and normal human tissues. **(A)** The expression level of NFE2L3 in different normal tissues was analyzed by the HPA and GTEx databases. **(B)** The CCLE database was used to analyze the expression levels of NFE2L3 in different cancer cell lines. **(C)** The TIMER2 database was used to analyze the expression levels of NFE2L3 in tumor and normal tissues. **(D)** TCGA database was used to analyze the expression levels of NFE2L3 in tumor tissues and adjacent normal tissues. ns, *p* ≥ 0.05; ∗, *p* < 0.05; ∗∗, *p* < 0.01; ∗∗∗, *p* < 0.001.

**FIGURE 2:**
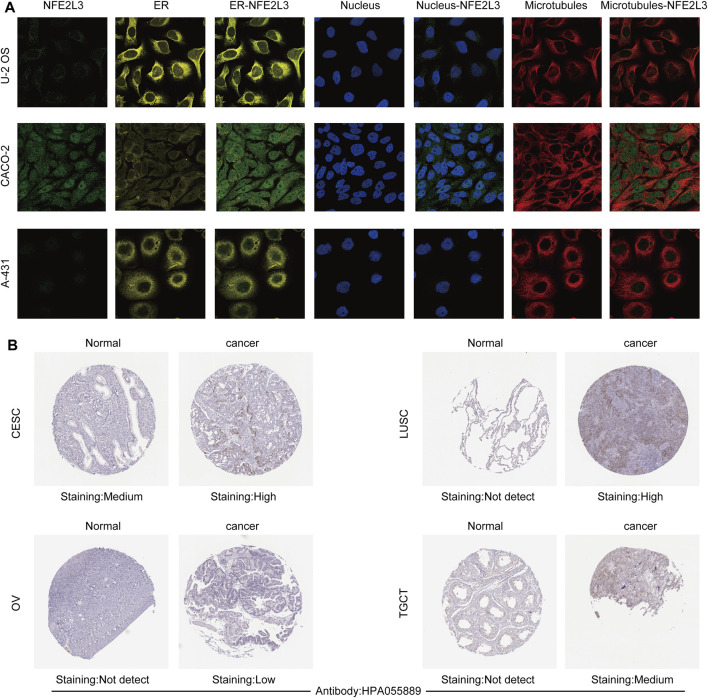
Subcellular localization and immunohistochemical analysis of NFE2L3. **(A)** NFE2L3 was mainly located in the nucleoplasm and vesicles by immunofluorescence (ICC-IF) and confocal microscopy. **(B)** The expression levels of NFE2L3 protein in four tumors and normal tissues were analyzed by immunohistochemistry. The results were all obtained from the HPA database.

The expression level of NFE2L3 in normal human tissues using the HPA database was evaluated. The results showed that the expression level of NFE2L3 was low in most normal tissues, however it was high in the retina, pancreas, and skin (Figure 1A). NFE2L3 was expressed at high levels in a variety of tumor cell lines, according to the CCLE database analysis (Figure 1B). The TIMER2.0 database analysis showed that the expression of NFE2L3 in 18 cancer types was significantly higher than that in normal tissues, including bladder urothelial carcinoma (BLCA), breast invasive carcinoma (BRCA), cervical squamous cell carcinoma endocervical adenocarcinoma (CESC), cholangiocarcinoma (CHOL), colon adenocarcinoma (COAD), esophageal carcinoma (ESCA), glioblastoma multiforme (GBM), head and neck squamous cell carcinoma (HNSC), KIRC, kidney renal papillary cell carcinoma (KIRP), LIHC, lung adenocarcinoma (LUAD), lung squamous cell carcinoma (LUSC), prostate adenocarcinoma (PRAD), rectum adenocarcinoma (READ), stomach adenocarcinoma (STAD), thyroid carcinoma (THCA), and uterine corpus endometrial carcinoma (UCEC) (Figure 1C). Furthermore, NFE2L3 expression was significantly upregulated in paired cancer and adjacent samples of 16 cancer types, including BLCA, BRCA, CHOL, COAD, ESCA, HNSC, KIRC, KIRP, LIHC, LUAD, LUSC, PRAD, READ, STAD, THCA, and UCEC (Figure 1D). NFE2L3 was also demonstrated to be localized in the nucleoplasm and vesicles (Figure 2A), and the protein expression level of NFE2L3 was significantly higher in ovarian serous cystadenocarcinoma (OV), TGCT, LUSC, and CESC than in normal tissues (Figure 2B). The above results demonstrated the upregulation of NFE2L3 expression in a variety of tumors, suggesting that NFE2L3 expression may promote tumor progression.

### Clinicopathological Correlation and Survival Prognostic Analysis

**FIGURE 3:**
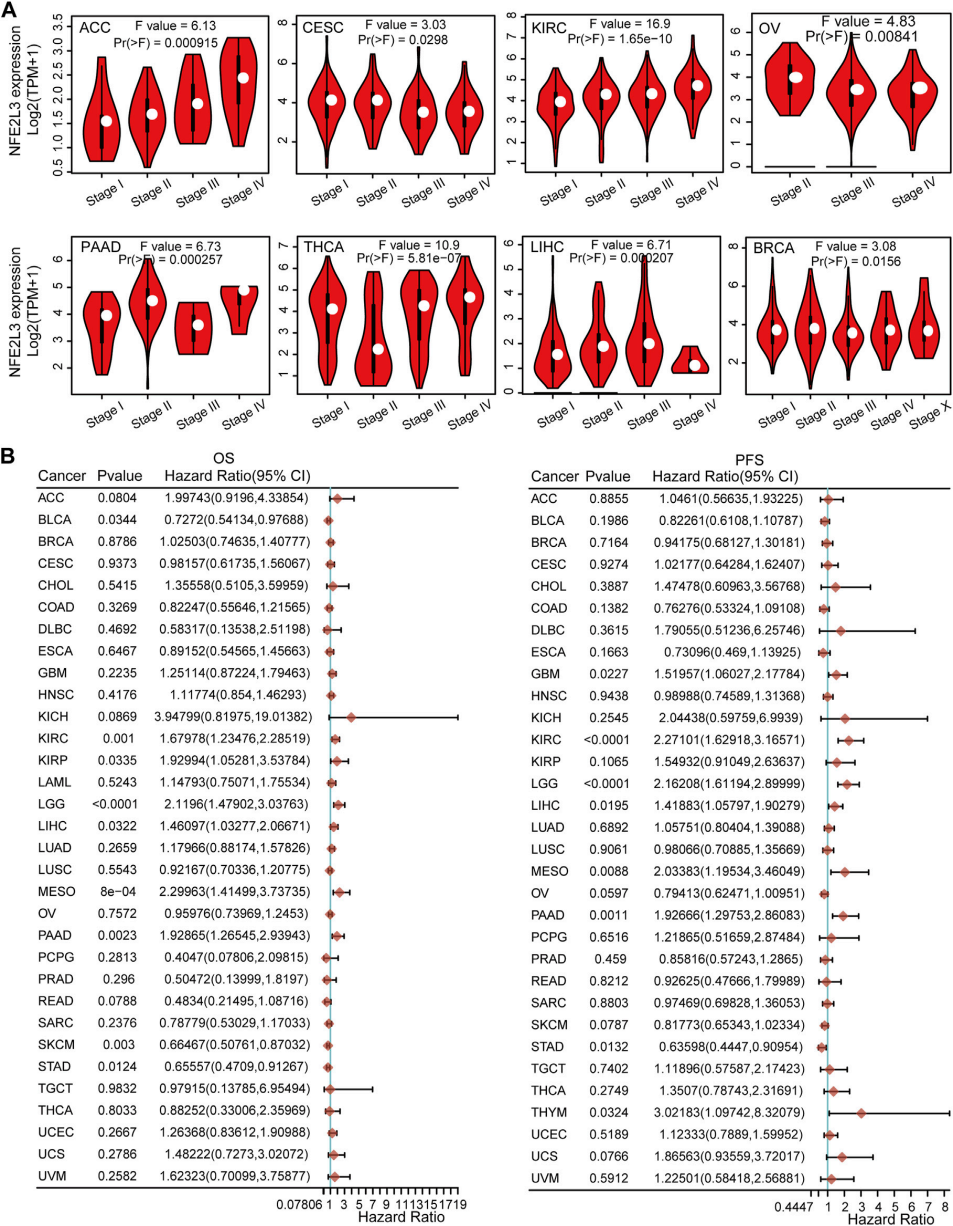
The relationship between NFE2L3 expression and prognosis and pathological stages in different tumors. **(A)** The association between NFE2L3 expression and the pathological stage was analyzed in ACC, CESC, KIRC, OV, PAAD, THCA, LIHC, and BRCA by the GEPIA2 database. **(B)** The correlation of NFE2L3 expression and Overall survival (OS) and progression-free survival (PFS) prognosis in pan-cancer.

The correlation between NFE2L3 expression and clinicopathological stage in pan-cancer was also investigated. Our study showed that NFE2L3 expression correlated with the clinicopathological stages (stages I, II, III, IV, and X) of eight tumors, including ACC, CESC, KIRC, OV, PAAD, THCA, LIHC, and BRCA (Figure 3A).

To further explore the relationship between NFE2L3 expression and prognosis, overall survival (OS), progression-free survival (PFS), and disease-free survival (DSS) analyses with median group cutoff by univariate Cox regression analysis for pan-cancers were performed. This study showed that higher expression of NFE2L3 had poorer prognosis for OS in six tumor types, including KIRC, KIRP, brain lower grade glioma (LGG), LIHC, mesothelioma (MESO), and pancreatic adenocarcinoma (PAAD). In contrast, higher expression of NFE2L3 was associated with a better prognosis for OS in BLCA, skin cutaneous melanoma (SKCM), and STAD. For PFS, higher expression of NFE2L3 had a poor prognosis in seven tumor types, including GBM, KIRC, LGG, LIHC, MESO, PAAD, and THYM. However, higher NFE2L3 expression was associated with a better prognosis for PFS in patients with STAD (Figure 3B). Furthermore, for DSS, the higher expression of NFE2L3 was associated with a worse prognosis in five tumor types, including KIRC, LGG, LIHC, MESO, and PAAD. However, higher NFE2L3 expression was associated with a better prognosis for DSS in COAD, SKCM, and STAD (Supplementary Figure S1). These results suggest that NFE2L3 expression is closely related to tumor progression and patient prognosis, thus NFE2L3 can be used as a prognostic marker for a variety of cancers.

### Diagnostic Value Analysis

**FIGURE 4:**
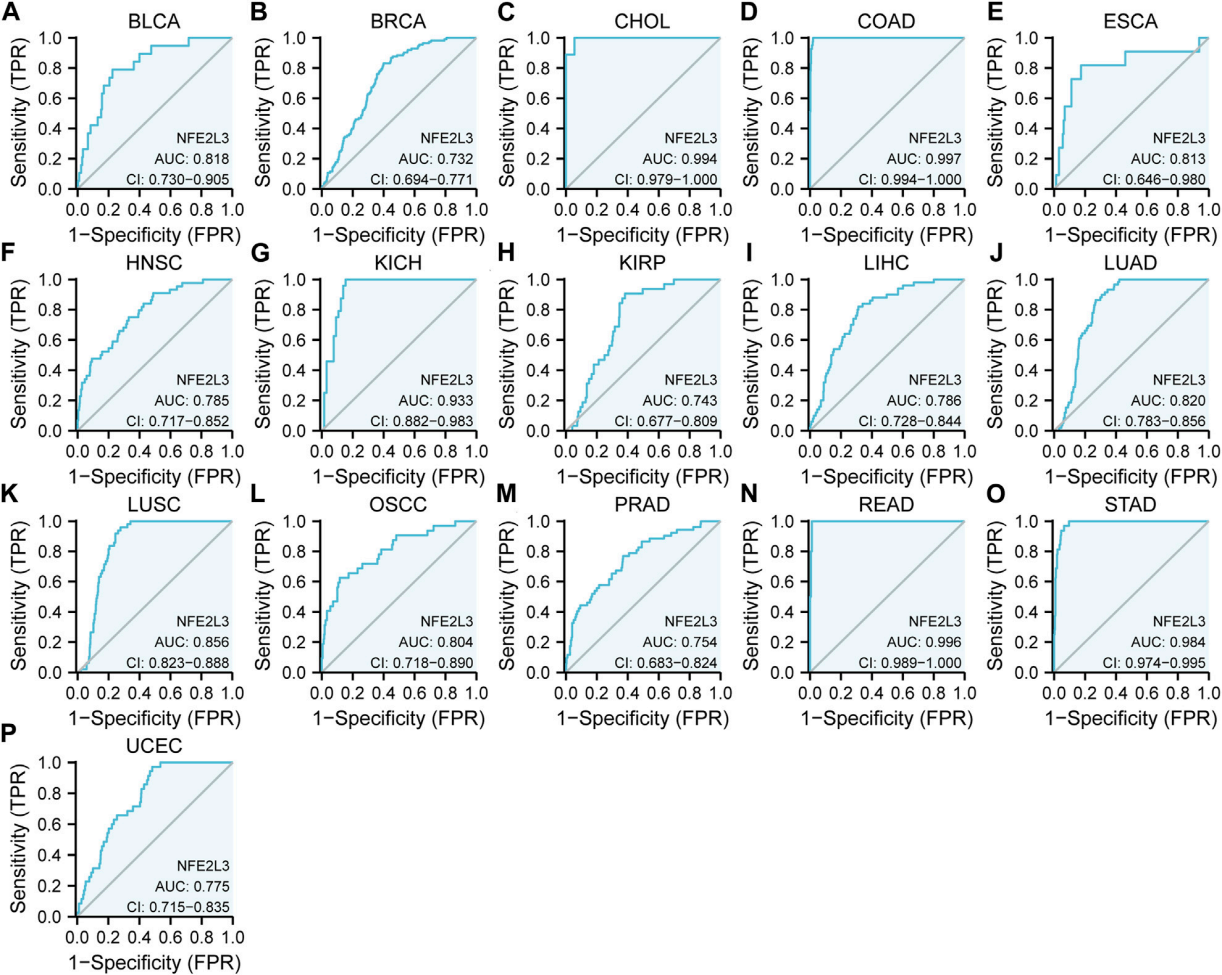
ROC curve of NFE2L3 expression levels in different tumors. **(A)** BLCA; **(B)** BRCA; **(C)** CHOL; **(D)** COAD; **(E)** ESCA; **(F)** HNSC; **(G)** KICH; **(H)** KIRP; **(I)** LIHC; **(J)** LUAD; **(K)** LUSC; **(L)** OSCC; **(M)** PRAD; **(N)** READ; **(O)** STAD; **(P)** UCEC.

The diagnostic value of NFE2L3 expression in pan-cancer was evaluated by using the ROC curve. As shown in Figures 4A–P, NFE2L3 had promising efficacy in the diagnosis of 11 tumors, including BLCA (AUC = 0.818), BRCA (AUC = 0.732), ESCA (AUC = 0.813), HNSC (AUC = 0.785), KIRP (AUC = 0.743), LIHC (AUC = 0.786), LUAD (AUC = 0.820), LUSC (AUC = 0.856), OSCC (AUC = 0.804), PRAD (AUC = 0.754), and UCEC (AUC = 0.775). Notably, NFE2L3 exhibited high accuracy in the diagnosis of five tumors, including CHOL (AUC = 0.994), COAD (AUC = 0.997), KICH (AUC = 0.933), READ (AUC = 0.996), and STAD (AUC = 0.984). This further demonstrated that NFE2L3 can be used as a diagnostic biomarker for a variety of tumors.

### DNA Methylation Analysis

**FIGURE 5:**
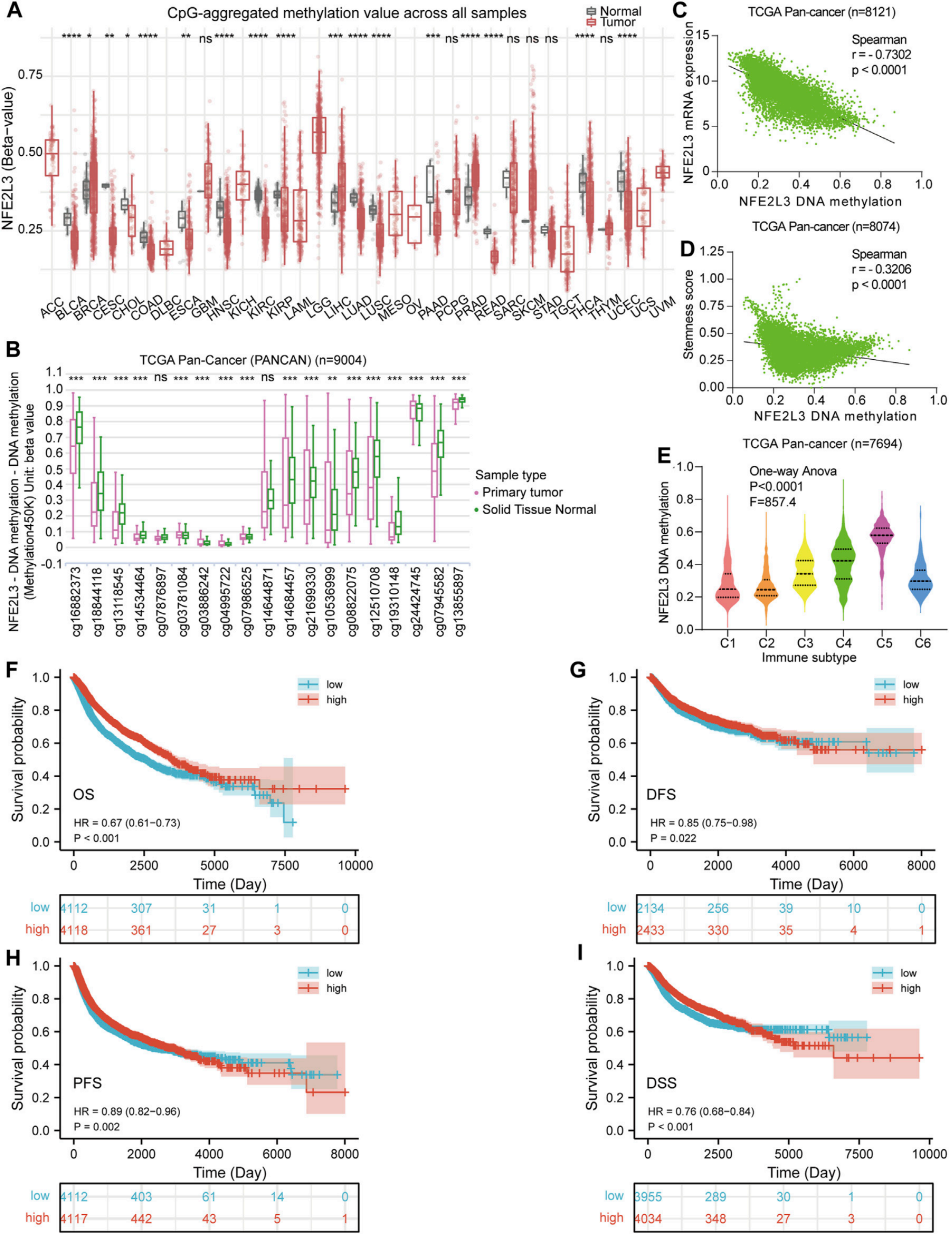
DNA methylation analysis of NFE2L3 in pan-cancer. **(A)**Analysis of DNA methylation levels of NFE2L3 in pan-cancer using the SMART database. **(B)** The 19 CPG methylation sites of NFE2L3 in TCGA pan-cancer (PANCAN) cohort were analyzed by the UCSC Xena database for comparison of NFE2L3 DNA methylation levels between tumor and normal groups. **(C)** The correlation between DNA methylation levels of NFE2L3 and NFE2L3 mRNA expression levels in TCGA PANCAN cohort. **(D)** The correlation between DNA methylation levels of NFE2L3 and tumor cell stemness in TCGA PANCAN cohort. **(E)** The DNA methylation levels of NFE2L3 in different immune subtypes in TCGA PANCAN cohort. **(F–I)** The association between DNA methylation levels of NFE2L3 and OS, disease-free survival (DFS), PFS, and disease-specific (DSS) prognosis in TCGA PANCAN cohort. ns, *p* ≥ 0.05; ∗, *p* < 0.05; ∗∗, *p* < 0.01; ∗∗∗, *p* < 0.001; ****, *p* < 0.0001.

Tumorigenesis and epigenetic modifications of genes are closely related, and DNA methylation is one of the most widely studied types of epigenetic modification. Thus, DNA methylation levels of NFE2L3 in normal and primary tumor tissues in pan-cancer were assessed. The DNA methylation levels of NFE2L3 were lower in BLCA, CESC, CHOL, COAD, ESCA, HNSC, KIRC, KIRP, PRAD, LUSD, LUSC, PAAD, READ, THCA, and UCEC than in normal tissues. However, the DNA methylation levels of NFE2L3 were higher in BRCA, LIHC, and PRAD tissues than in normal tissues (Figure 5A). In addition, we showed that the DNA methylation levels of NFE2L3 were significantly lower in tumor tissues than in normal tissues in most of the NFE2L3 methylation CpG sites in TCGA pan-cancer (PANCAN) cohort, including cg16882373, cg18844118, cg13118545, cg14534464, cg03886242, cg04995722, cg07986525, cg14684457, cg21699330, cg10536999, cg08822075, cg12510708, cg19310148, cg07945582, and cg13855897 (Figure 5B). There was a significant negative correlation between the DNA methylation level of NFE2L3, the mRNA expression level and tumor cell stemness score in TCGA PANCAN cohort (Figures 5C,D). DNA methylation levels of NFE2L3 significantly correlated with immune subtypes in TCGA PANCAN cohort, with lower DNA methylation levels of NFE2L3 in the immune subgroups of C1 and C2 types, however higher DNA methylation levels of NFE2L3 in the immune subgroup of C5 types were observed (Figure 5E). This study demonstrated that lower NFE2L3 DNA methylation levels were associated with worse OS, PFS, DSS, and disease-free survival (DFS) prognosis in TCGA PANCAN cohort (Figures 5F–I).

### Genetic Alteration Analysis

**FIGURE 6:**
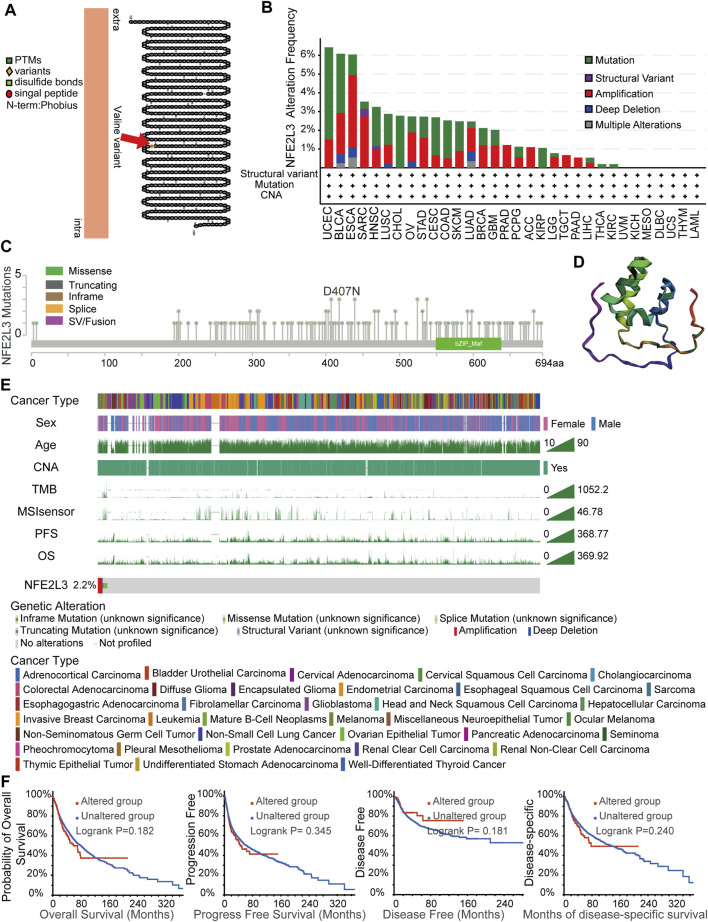
NFE2L3 variant and the characteristics of NFE2L3 mutations in TCGA cohort. **(A)** A natural missense variant of Valine441 in transmembrane protein topology of NFE2L3. **(B)** Genetic alteration frequencies of NFE2L3 in TCGA pan-cancer cohorts. **(C)** Type and site of mutations of NFE2L3. **(D)** Variant distribution of 3D protein structure of NFE2L3. **(E)** The correlation of NFE2L3 status and clinical characteristics in TCGA pan-cancer cohort. **(F)** The correlation between NFE2L3 mutation status and prognosis (OS, PFS, DFS, and DSS) of cancer.

Accumulation of gene mutations is one of the causes of tumorigenesis; therefore, the landscape of genetic alterations of NFE2L3 in pan-cancer were analyzed. Transmembrane protein topology of NFE2L3 revealed a natural missense variant of Valine441 in membrane localization (Figure 6A). The genetic alteration status of NFE2L3 in multiple tumor samples from the TCGA PANCAN cohort were investigated using the cBioPortal. As shown in Figure 6B, NFE2L3 mutations occurred in most tumor types, with the top three tumors, UCEC, BLCA, and ESCA, having high NFE2L3 mutations (>6%). The tumor, UCEC, had the highest mutation of NFE2L3 (>6%). A total of 139 NFE2L3 mutations were identified, 108 (77.7%) were missense mutations, 25 (18.0%) were truncating mutations, 3 (2.2%) were fusion mutations, 2 (1.4%) were in-frame mutations, and 1 (0.7%) was a splice mutation (Figure 6C). All mutations were dispersed in the full sequence and 3D protein structure of NFE2L3 (Figures 6C,D). As shown in Figure 6E, genetic alterations in NFE2L3 were associated with multiple clinical characteristics. The overall alteration frequency of NFE2L3 was 2.2% (244/10,950) in TCGA pan-cancer cohort. In addition, we performed a survival analysis of the genetic alteration status of NFE2L3 was performed on the entire TCGA pan-cancer cohort, however the results revealed no significant correlation between the genetic alteration status of NFE2L3 and patient prognosis. (Figure 6F).

### Immune Feature and Immunotherapy Response Analysis

**FIGURE 7:**
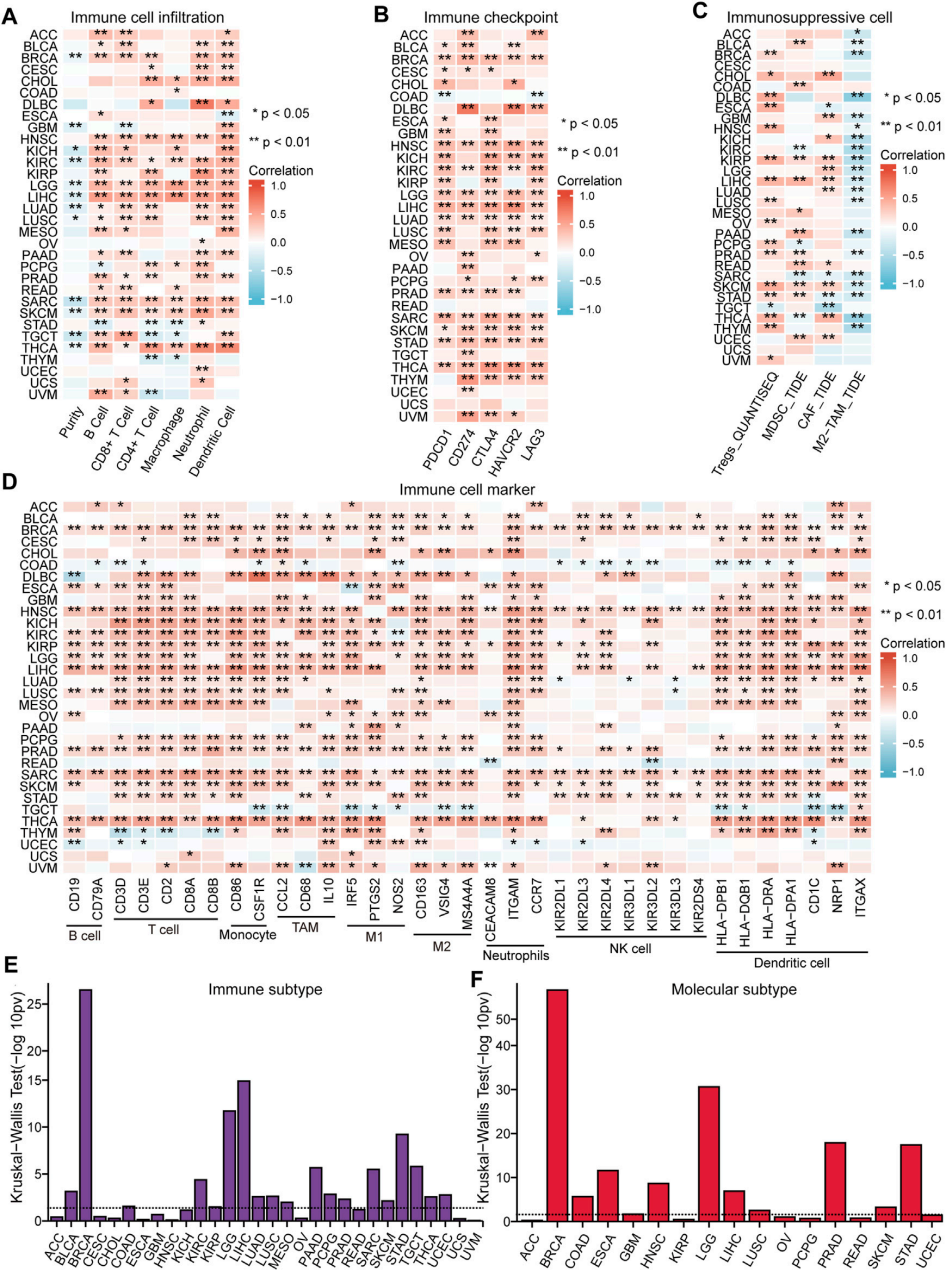
The correlation between NFE2L3 expression and immune feature in pan-cancer. **(A)** The correlation between NFE2L3 expression and immune infiltration in pan-cancer. **(B)** The correlation between NFE2L3 expression and immune checkpoint in pan-cancer. **(C)** The correlation between NFE2L3 expression and T cell exclusion filtration in pan-cancer. **(D)** The correlation between NFE2L3 expression and immune cell marker in pan-cancer. **(E)** The correlation between NFE2L3 expression and immune subtype in pan-cancer. **(F)** The correlation between NFE2L3 expression and molecular subtype in pan-cancer.

The immune response of tumors is an important process in tumor development, thus the role of NFE2L3 in tumor immune regulation was investigated by analyzing the immune features and immunotherapeutic responses. The correlations between the expression of NFE2L3 and different immune signatures in TCGA pan-cancer cohort were evaluated, including immune cell infiltration, immune checkpoints, immunosuppressive cell infiltration, and immune cell markers. A significant positive correlation between the expression of NFE2L3 and the level of immune cell infiltration in TCGA pan-cancer cohort was demonstrated, except for COAD, OV, UCEC, and UCS (Figure 7A). However, a significant positive correlation between NFE2L3 expression and immune checkpoint expression in TCGA pan-cancer cohort was demonstrated except for READ, UCEC, and UCS (Figure 7B). A significant positive correlation was also shown between NFE2L3 expression and the levels of Tregs, MDSC, and CAF in TCGA pan-cancer cohort, except for ACC, CESC, and UCS. However, NFE2L3 expression and the level of M2-TAM infiltration were significantly negatively correlated in most tumors (Figure 7C). Our study showed a significant positive correlation between NFE2L3 expression and most immune cell markers in TCGA pan-cancer cohort, except for UCS (Figure 7D). This study showed that NFE2L3 expression was also significantly positively correlated with three immunomodulators, including immunoinhibitory, immunostimulatory, and MHC molecules in TCGA pan-cancer cohort (Supplementary Figure S2). The correlations between NFE2L3 expression and immune or molecular subtypes in TCGA pan-cancer cohort from the TISIDB database were investigated. The results showed that NFE2L3 was expressed differently in different immune subtypes of 12 cancer types, including KIRC, COAD, BRCA, UCEC, STAD, SKCM, PRAD, PAAD, MESO, LUSC, LIHC, and LGG (Figure 7E). The differential expression of NFE2L3 was also found in various molecular subtypes of the nine cancer types, including UCEC, STAD, PRAD, LUSC, LIHC, LGG, HNSC, COAD, and BRCA (Figure 7F).

**FIGURE 8:**
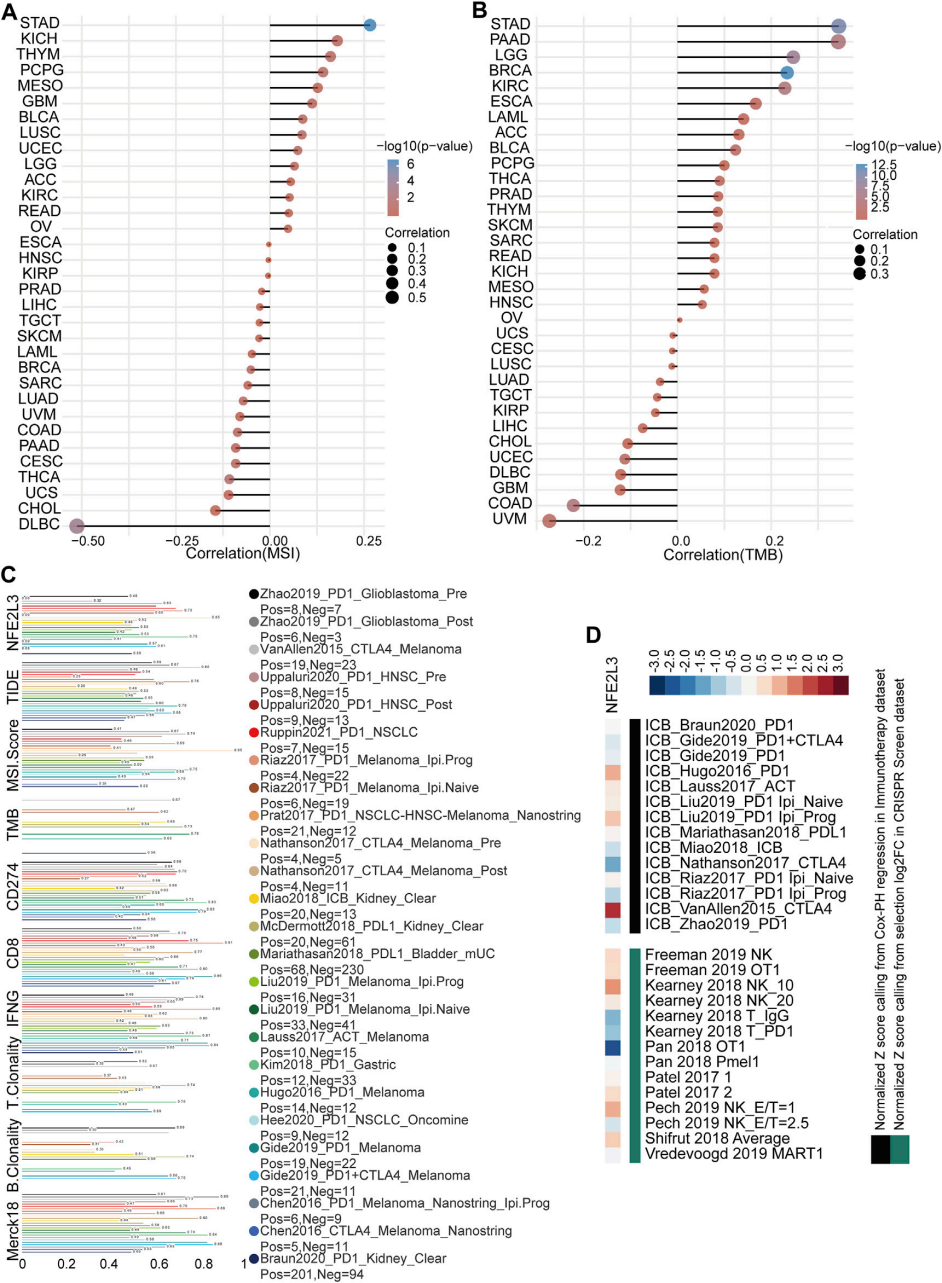
Immunotherapy response analysis in pan-cancer. **(A)** The correlation between NFE2L3 expression and MSI in pan-cancer. **(B)** The correlation between NFE2L3 expression and TMB in pan-cancer. **(C)** The potential of NFE2L3 as a biomarker to predict response to immune checkpoint blockade. **(D)** NFE2L3 was ranked by its weighted average value across the association with ICB survival outcome and log-fold change (logFC) in CRISPR screens.

The effect of NFE2L3 expression on immunotherapy was evaluated by analyzing the correlation between NFE2L3 expression and TMB or MSI. The results showed that NFE2L3 expression was significantly positively correlated with MSI in STAD, KICH, and THYM, however NFE2L3 expression was negatively correlated with DLBC and CHOL (Figure 8A). NFE2L3 expression was significantly positively correlated with TMB in STAD, PAAD, LGG, BRCA, and KIRC however it was negatively correlated with COAD and UVM (Figure 8B). The potential of NFE2L3 as a biomarker to predict the response to immune checkpoint blockade (ICB) was evaluated. The results showed that NFE2L3 had a limited accuracy (AUC > 0.5) in predicting the response to ICB in 12 of the 23 ICB sub-cohorts and compared with TMB, T cell clonality, and B cell clonality, NFE2L3 displayed a higher predictive power (Figure 8C). High NFE2L3 expression was associated with poor survival prognosis in the ICB_VanAllen2015_CTLA4, ICB_Hugo2016_PD1, and ICB_Liu2019_PD1 Ipi_Prog cohorts. However, knockdown of NFE2L3 enhanced the efficacy of lymphocyte-mediated tumor killing in the Kearney 2018 NK_10, Pech 2019 NK_E/T = 1, and Shifrut 2018 average cohorts (Figure 8D). These results suggested that NFE2L3 may promote tumor development by regulating tumor immunity.

### Single Cell Sequencing Analysis

**FIGURE 9:**
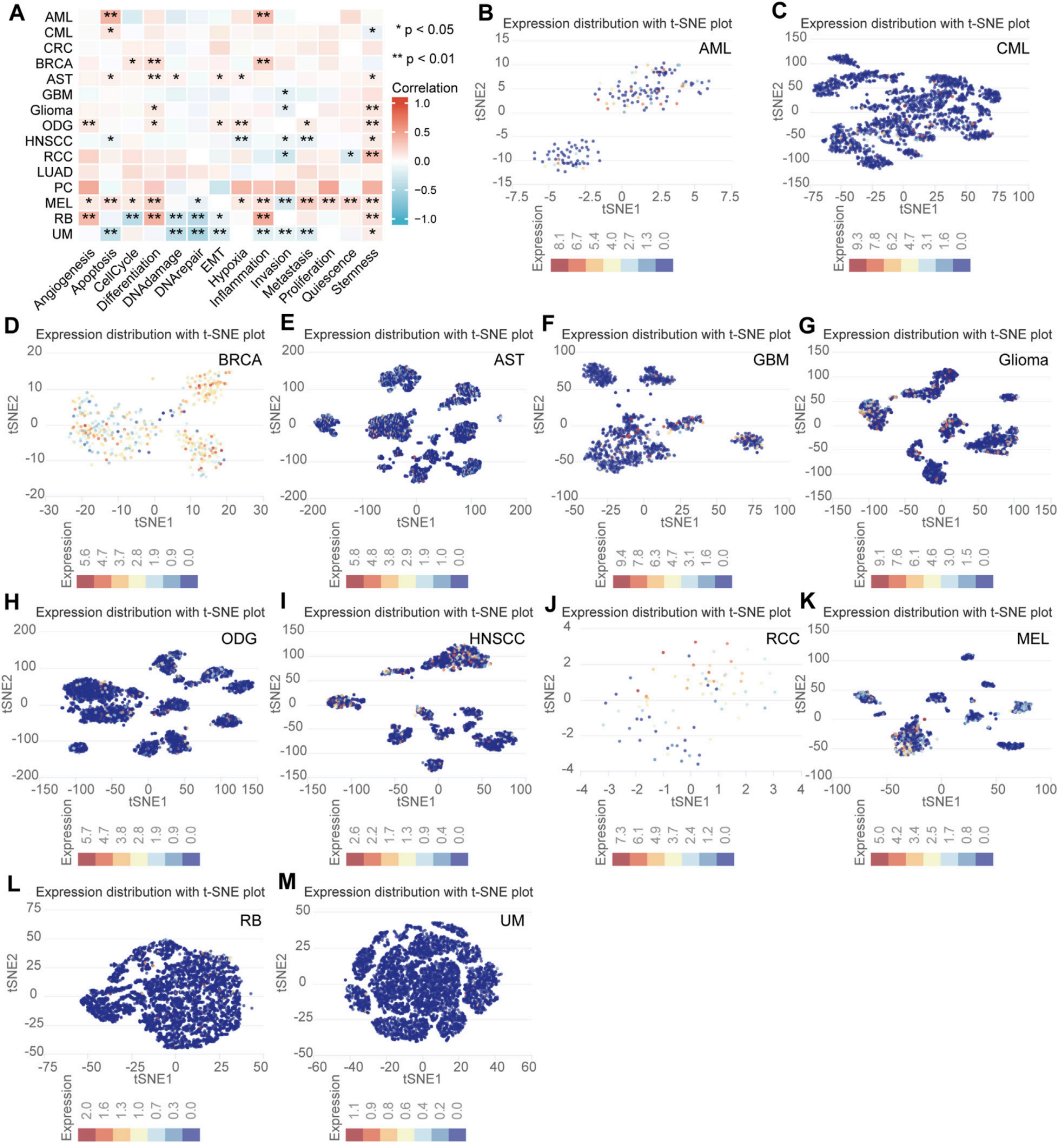
The correlation analysis of NFE2L3 expression and single cell functional status in multiple tumors. **(A)** Heat map of correlation between NFE2L3 expression and single cell function in multiple tumors. **(B–M)** T-SNE diagram of NFE2L3 expression levels in single cells of multiple tumors including acute myeloid leukemia (AML), chronic myelogenous leukemia (CML), breast cancer (BRCA), astrocytoma (AST), glioblastoma (GBM), glioma, oligodendroglioma (ODG), head and neck cancer (HNSCC), renal cell carcinoma (RCC), melanoma (MEL), retinoblastoma (RB), and uveal melanoma (UM).

The expression level and functional status of NFE2L3 in single tumor cells using the CancerSEA database was determined. NFE2L3 expression was shown to be closely related to the cellular functional status of a variety of tumors, including acute myeloid leukemia (AML), chronic myelogenous leukemia (CML), breast cancer (BRCA), astrocytoma (AST), glioblastoma (GBM), glioma, oligodendroglioma (ODG), head and neck cancer (HNSCC), renal cell carcinoma (RCC), melanoma (MEL), retinoblastoma (RB), and uveal melanoma (UM). In most tumor cells, NFE2L3 expression was positively correlated with differentiation, angiogenesis, apoptosis, inflammation, and tumor cell stemness however, NFE2L3 expression negatively correlated with DNA damage, DNA repair, and invasion (Figure 9A). NFE2L3 was expressed at higher levels in single cells of AML, CML, BRCA, AST, GBM, glioma, ODG, RCC, and MEL monocyte samples however, it was expressed at lower levels in single cells of HNSCC, RB, and UM samples (Figures 9B–M).

### Gene Function Enrichment Analysis

**FIGURE 10:**
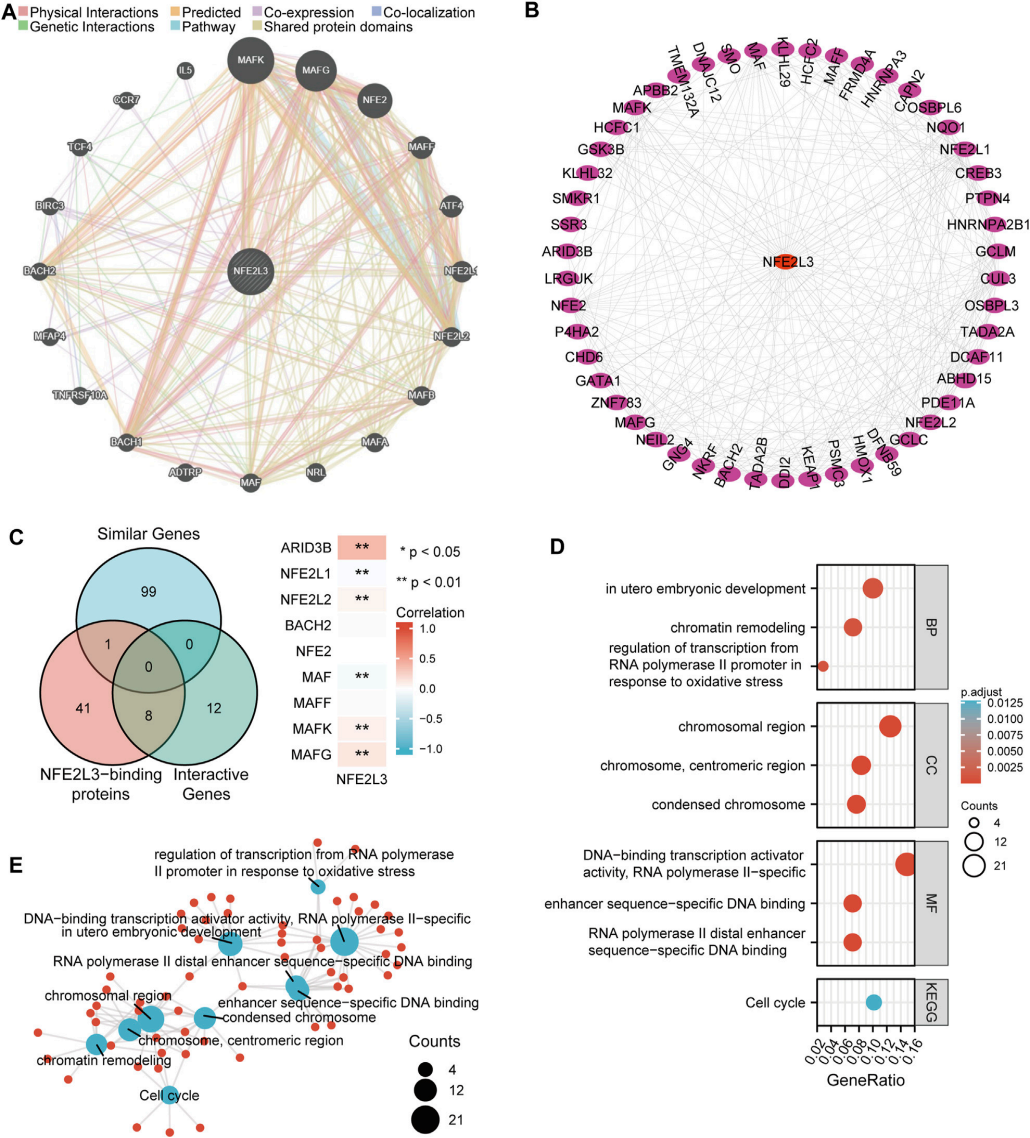
Functional enrichment analysis of NFE2L3-related genes. **(A)** Top 20 interactive genes of NFE2L3. **(B)** Top 50 NFE2L3-binding proteins. **(C)** Venn diagram of NFE2L3-related genes and the correlation between expression levels of NFE2L3 and nine overlapping genes. **(D,E)** GO and KEGG pathway enrichment analysis of NFE2L3-related genes.

The molecular functions of NFE2L3 was evaluated in different tumors by performing GO and KEGG pathway enrichment analyses. The top 20 genes that interacted with NFE2L3 were evaluated using the GeneMANIA database (Figure 10A). Then the top 50 proteins interacting with NFE2L3 were predicted and visualized using the STRING database using Cytoscape, respectively (Figure 10B). The GEPIA2 database was then used to explore the top 100 genes with a similar expression pattern to NFE2L3 in the pan-cancer analysis. The intersection of NFE2L3-related genes predicted by the above three databases were used and the results were visualized using a Venn diagram. Nine genes were predicted by at least two databases, including NFE2L1, NFE2L2, BACH2, NFE2, MAF, MAFF, MAFK, MAFG, and ARID3B. Furthermore, the expression of NFE2L3 was shown to be significantly correlated with the expression of six genes at the intersection in TCGA pan-cancer cohort by the GEPIA2 database (Figure 10C). In addition, GO and KEGG pathway enrichment analyses of NFE2L3-related genes using the three databases were performed. GO enrichment analysis revealed that the primary biological process (BP) was mainly related to embryonic development and regulation of transcription from the RNA polymerase II promoter in response to oxidative stress and chromatin remodeling. The cellular component (CC) contained chromosomal regions, chromosomes, centromeric regions, and condensed chromosomes. The molecular function (MF) was primarily enriched in DNA-binding transcription activator activity, RNA polymerase II-specific, RNA polymerase II distal enhancer sequence-specific DNA binding, and enhancer sequence-specific DNA binding. The KEGG pathway enrichment was involved in the cell cycle (Figures 10D,E).

GSEA analysis in BRCA, CHOL, ESCA, HNSC, KIRC, PRAD, UCEC, and THCA was performed. Supplementary Figure S3 showed the top 10 enriched signaling pathways in each tumor according to the normalized enrichment score (NES). NFE2L3 was involved in CD22 mediated BCR regulation, FCGR activation, and creation of C4 and C2 activators in most tumors. NFE2L3 was also involved in the role of LAT2 calcium and the role of phospholipids in phagocytosis in BRCA, CHOL, THCA, PRAD, and UCEC. NFE2L3 was shown to be related to immunoregulatory interactions between lymphoid and non-lymphoid cells in CHOL, KIRC, and THCA. NFE2L3 was shown to be associated with FCGR3A mediated IL10 synthesis in KIRC, PRAD, and THCA. These results also demonstrated that NFE2L3 played a key role in tumor immunomodulatory regulation. In addition, NFE2L3 was observed to be involved in vitamin B12 metabolism and in the statin pathway in ESCA. NFE2L3 was related to the ribosome in HNSC and involved in the scavenging of heme from plasma in THCA.

### Co-Expression Gene and Functional Enrichment Analysis of NFE2L3 in Liver Hepatocellular Carcinoma

**FIGURE 11:**
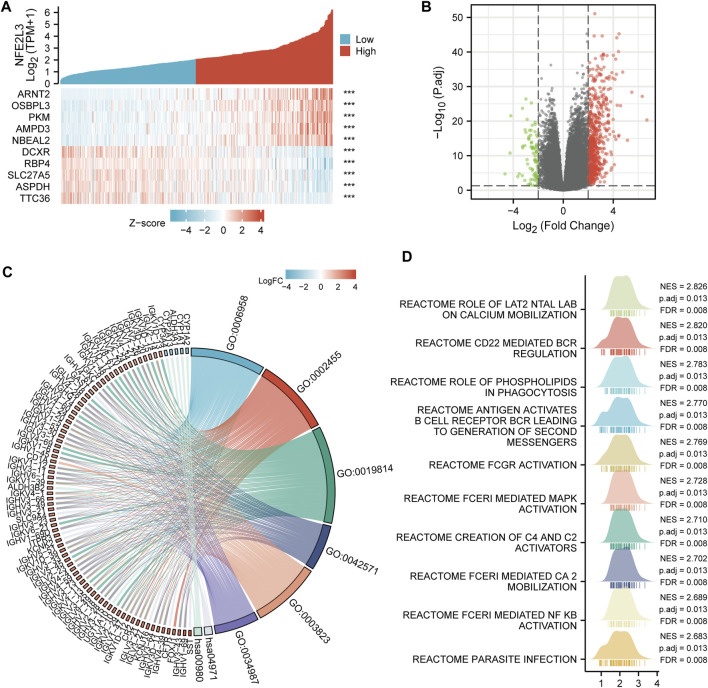
Co-expression gene and functional enrichment analysis of NFE2L3 in LIHC. **(A)** The top five genes positively correlated with NFE2L3 expression, and the top five genes negatively correlated with NFE2L3 expression in LIHC. **(B)** The volcano map of DEGs between NFE2L3 high expression group and low expression groups in LIHC. **(C)** GO and KEGG pathway enrichment analyses of DEGs. **(D)** GSEA merged plots indicated the top 10 significant signaling pathways associated with NFE2L3 expression according to Reactome analyses in LIHC.

The correlation between NFE2L3 expression and LIHC was examined. The top five co-expression genes that were positively correlated with NFE2L3 expression in LIHC, including ARNT2, OSBPL3, PKM, AMPD3, and NBEAL2, were evaluated. The top five co-expression genes that negatively correlated with NFE2L3 expression in LIHC, including DCXR, RBP4, SLC27A5, ASPDH, and TTC36, were also evaluated (Figure 11A). The DEGs were analyzed between the NFE2L3 high and low expression groups in LIHC with threshold values of |log2 FC| > 2.0, and adjusted *p*-value < 0.05. We identified 749 DEGs, including 668 upregulated and 81 downregulated genes (Figure 11B). Furthermore, GO and KEGG pathway enrichment analyses were performed on the DEGs that met the screening requirements. The results indicated that BP was related to the classical complement activation pathway and humoral immune response mediated by circulating immunoglobulin. The CC consisted of immunoglobulin complexes and circulating immunoglobulin complexes. The MF was primarily enriched in antigen and immunoglobulin receptor binding. The KEGG pathway enrichment was involved in gastric acid secretion and the metabolism of xenobiotics by cytochrome P450 (Figure 11C). In addition, GSEA analysis was performed in LIHC, and the top 10 significant terms of GSEA were mainly involved in immune-related pathways, metabolic functions, and parasite infection (Figure 11D).

### Prognostic Model Based on NFE2L3 and Clinical Characteristics in Liver Hepatocellular Carcinoma

**FIGURE 12:**
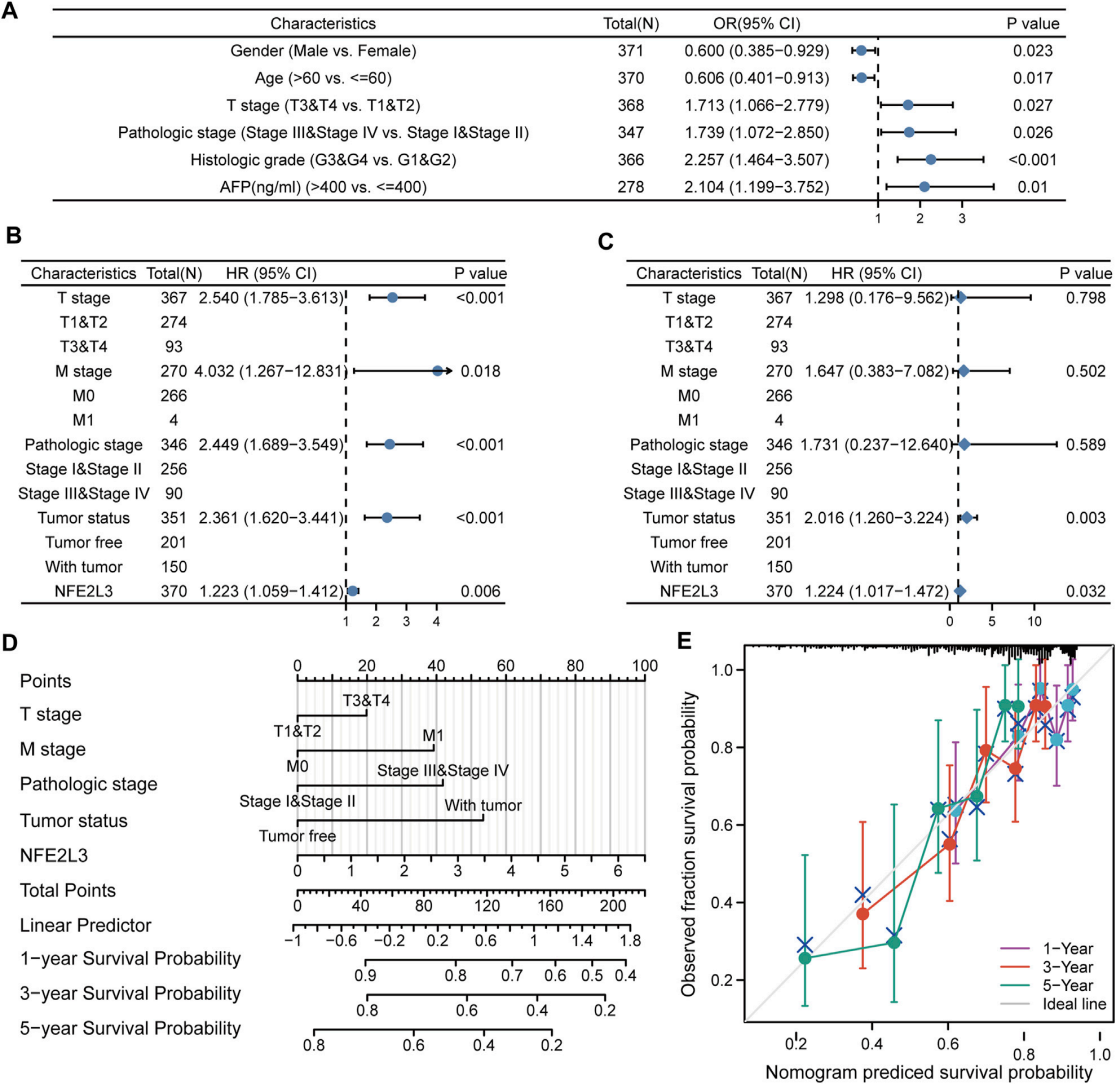
Relationship between the expression level of NFE2L3 and clinical characteristics in LIHC. **(A)** Logistic regression analysis of expression level and clinical characteristics of NFE2L3 in LIHC. **(B,C)** Forest plots of the univariate and multivariate Cox in LIHC. **(D)** A nomogram based on T stage, M stage, pathologic stage, tumor status, and NFE2L3 for predicting the OS of patients with LIHC. **(E)** The calibration plot indicated the calibration of the nomogram model.

The relationship between NFE2L3 and clinical characteristics of patients with LIHC was evaluated. The baseline clinical characteristics of the patients in TCGA-LIHC cohort based on the expression levels of NFE2L3 were analyzed (Supplementary Table S1). The correlation between NFE2L3 expression levels and the clinical characteristics of the patients were evaluated using logistic regression analysis. The results indicated that high NFE2L3 expression was significantly correlated with sex, age, T stage, pathologic stage, histologic grade, and AFP (Figure 12A). Moreover, univariate Cox regression analysis revealed that the OS of patients with LIHC was significantly correlated with T stage, M stage, pathological stage, tumor status, and NFE2L3 expression (Figure 12B). Multivariate Cox regression analysis further demonstrated that NFE2L3 expression was an independent prognostic factor for OS in LIHC patients (HR = 1.224, 95% CI = 1.017–1.472, *p* = 0.032) (Figure 12C). In addition, a nomogram model using T stage, M stage, pathological stage, tumor status, and NFE2L3 expression was constructed to predict the OS of patients in TCGA-LIHC cohort at one, three, and 5 years (Figure 12D) The calibration curve demonstrated that the model had good calibration (Figure 12E).

## Discussion

Cancer is one of the greatest threats to human health worldwide, with a high incidence and mortality rate (Sung et al., 2021). Early diagnosis and treatment of cancer is crucial for patient prognosis. Pan-cancer analysis can reveal tumor-specific and common molecular signatures, identify novel biomarkers, and provide new insights into the development of effective prevention and therapeutic strategies for human cancers (Chen et al., 2021). NFE2L3 belongs to the Cap n’ Collar basic-region leucine zipper family of transcription factors. The NFE2L3 protein is a membrane-bound glycoprotein that targets the endoplasmic reticulum and the nuclear envelope. Recent studies have confirmed that NFE2L3 is linked to certain cancers in humans, including colon cancer, bladder cancer, breast cancer, pancreatic cancer, and liver hepatocellular carcinoma (Wang et al., 2018; Bury et al., 2019; Ren et al., 2020; Dai et al., 2021; Qian et al., 2022). NFE2L3 plays a role in transcription of many biological processes, confers selective growth advantages to cells, and promotes cancer progression, including proliferation, invasiveness, metastasis, and angiogenesis (Kobayashi, 2020).

However, no studies to our knowledge have evaluated the significance of NFE2L3 expression in pan-cancer analyses. The molecular mechanisms of NFE2L3 in cancer was evaluated by performing a comprehensive analysis of NFE2L3 in 33 types of human tumors based on data from the TCGA, CCLE, and HPA databases, including gene expression, epigenetic methylation, genetic alteration, functional enrichment, immune features, and survival prognosis. Our results showed that NFE2L3 was significantly upregulated in most human tumors including BLCA, BRCA, CESC, CHOL, COAD, ESCA, GBM, HNSC, KIRC, KIRP, LIHC, LUAD, LUSC, PRAD, READ, STAD, THCA, and UCEC. Furthermore, NFE2L3 protein was shown to be upregulated in OV, TGCT, LUSC, and CESC. NFE2L3 expression was significantly correlated with different pathological stages in ACC, CESC, KIRC, OV, PAAD, THCA, LIHC, and BRCA. In addition, upregulated expression of NFE2L3 was significantly associated with poor OS, DFS, and DSS in most tumors, including KIRC, KIRP, LGG, LIHC, MESO, PAAD, GBM, and THYM. NFE2L3 also had promising efficacy in tumor diagnosis, especially in the diagnosis of CHOL, COAD, KICH, READ, and STAD. Thus, our study demonstrated that NFE2L3 expression was upregulated in a variety of cancers and was associated with poor prognosis, consistent with previous reports (Wang et al., 2018; Bury et al., 2019; Dai et al., 2021; Wang et al., 2021; Qian et al., 2022). The above results suggest that NFE2L3 expression was closely related to tumor development and that NFE2L3 can be used as a new biomarker for diagnosis and prognosis in most tumors.

DNA methylation is a common epigenetic modification that is closely related to tumorigenesis, regulating gene transcription and expression, and has become the focus in epigenetics (Mehrmohamadi et al., 2016). Hypermethylation in the promoter region of tumor suppressor genes inhibits the expression of tumor suppressor genes and causes them to lose their tumor suppressor functionality, thus promoting the occurrence of cancer. Hypomethylation of the whole genome or of the proto-oncogene promoter, reduces the stability of the chromosome structure or activates proto-oncogenes, thus inducing cell carcinogenesis (Das and Singal, 2004). Our study demonstrated that the DNA methylation levels of NFE2L3 were downregulated in most tumors and negatively correlated with the expression levels of NFE2L3 mRNA and the tumor cell stemness score. The methylation level of NFE2L3 was significantly correlated with different immune subtypes, suggesting that the methylation epistasis regulation of NFE2L3 may be involved in tumor immunity. Moreover, survival analysis showed that hypermethylation of NFE2L3 was associated with good prognosis in TCGA pan-cancer (PANCAN) cohort. Wang et al. (2019) demonstrated that NFE2L3 was a novel DNA methylation driver gene and prognostic marker in human clear cell RCC. In contrast to the study by JW et al., our study was not limited to individual tumors, but explored the expression levels of NFE2L3 at each methylation site and the total methylation levels in pan-cancers. In addition, the correlation between NFE2L3 methylation levels and NE2L3 mRNA expression levels, tumor stemness, immune subtypes, and pan-cancer prognosis was evaluated. NFE2L3 might function as a proto-oncogene, and that downregulation of DNA methylation of NFE2L3 promoted upregulation of NFE2L3 expression in most tumors. The overexpression of NFE2L3 may promote tumorigenesis and progression by regulating tumor cell stemness and immune responses. However, the relationship between NFE2L3 DNA methylation and tumor progression requires further verification.

Previous studies have shown that tumor development is closely related to genetic alterations, especially mutations in oncogenes and tumor suppressor genes (Cohen et al., 2017; Liu et al., 2020b). Our results showed a natural missense variant of Valine441 in membrane localization. The total genetic alteration frequency of NFE2L3 was 2.2% (244/10,950) in TCGA pan-cancer cohort, and the highest alteration frequency of NFE2L3 was approximately 6.4% in patients with UCEC. Mutations and amplifications were the most common types of genetic alterations, with missense mutations being the main type, accounting for 77.7% of the mutations. However, there was little correlation between genetic alterations in NFE2L3 and patient prognosis.

With the continuous development of biomedical technology, tumor immunotherapy has become the fourth most effective treatment method after surgery, chemotherapy, and radiotherapy (Dou et al., 2022; Saliba et al., 2022). The potential of NFE2L3 in tumor immunotherapy was explored further by conducting a correlation analysis between the expression of NFE2L3 and immune characteristics. This study showed that NFE2L3 expression was significantly correlated with various immune characteristics in TCGA pan-cancer, including immune cell infiltration, immune checkpoints, immunosuppressive cell infiltration, immune cell markers, immunomodulators, immune subtypes, and molecular subtypes. In addition, NFE2L3 expression levels were significantly positively correlated with MSI and TBM in STAD. Our study also demonstrated that NFE2L3 expression was associated with patient survival in multiple ICB cohorts, and that knockdown of NFE2L3 improved the effect of ICB treatment for colon cancer in the Kearney 2018 NK_10 cohort. In conclusion, NFE2L3 shows potential in tumor immunotherapy and may be a new target for tumor immunotherapy.

Recently, single-cell sequencing technology has developed rapidly, and its most important feature is the ability to sequence the genome at the individual cell level compared to traditional sequencing technologies, thus enabling further exploration of patterns of tumor heterogeneity (Suvà and Tirosh, 2019). CancerSEA is a database based on single-cell sequencing and is used to analyze the different functional states of tumor cells at the single-cell level, facilitating further investigation of the functional heterogeneity of tumor cells (Yuan et al., 2019). NFE2L3 was expressed at high levels in most tumor cells according to the CancerSEA analysis and was closely associated with various tumor cell functional states, such as apoptosis, differentiation, inflammation, and stemness. This suggests that NFE2L3 may play an essential role in tumor development.

The molecular function of NFE2L3 in pan-cancer was investigated through gene function enrichment analysis. Our study showed that NFE2L3 was mainly involved in cell cycle regulation by KEGG pathway enrichment analysis, which was consistent with the study performed by Bury et al. (2019). NFE2L3 was observed to be involved in transcriptional regulation and chromatin remodeling of the RNA polymerase II promoter in response to oxidative stress by GO enrichment analysis. Moreover, Saliba et al. (2022) found that knockdown of NFE2L3 could prevent inflammation-induced colorectal cancer by regulating the tumor microenvironment. NFE2L3 was identified to be involved in multiple immune pathways in most tumors using GSEA analysis, including: CD22 mediated BCR regulation, FCGR activation, and the creation of C4 and C2 activators. YC et al. found that NFE2L3 was involved in metastasis and drug resistance in breast cancer. The signaling pathways related to drug resistance and metastasis were absent in our analysis, which may explain the differences in databases and the different thresholds chosen for the analysis.

The function of NFE2L3 was investigated by analyzing the co-expression of genes of NFE2L3 and DEGs between the NFE2L3 high and low expression groups in LIHC. Subsequently, GO and KEGG pathway enrichment analyses and GSEA on these DEGs were conducted and it was shown that they were mainly enriched in immune and metabolic processes. Our study also investigated the relationship between the expression level of NFE2L3 and clinicopathological features using logistic regression analysis and the clinical characteristics related to OS of LIHC using univariate and multivariate Cox regression analyses. NFE2L3 expression level correlated with sex, age, T stage, pathologic stage, histologic grade, and AFP in LIHC, and NFE2L3 expression was an independent risk factor for OS in patients with LIHC, which was consistent with previous studies (Yu et al., 2019; Ren et al., 2020). These results show that NFE2L3 may be a marker for identifying early LIHC and advanced LIHC. Furthermore, a prognostic nomogram using T stage, M stage, pathological stage, tumor status, and NFE2L3 was constructed to predict the one-, three-, and five-year OS in LIHC, which aids physicians in identifying patients at high risk for LIHC.

There are some limitations to the present study. Only online public databases were used for analysis, which may cause systematic bias. The study lacked actual clinical data and further experimental validation using cells and animal models to prove the results observed (Wei et al., 2019).

## Conclusion

Recently, with the rapid development of bioinformatics, an increasing number of computing methods have been developed and used in tumor research to promote tumor diagnosis and treatment technology (Anashkina et al., 2021; Rogers et al., 2021). To investigate the molecular function of NFE2L3 in pan-cancer, we performed a comprehensive and integrated analysis of NFE2L3 by combining multiple bioinformatic approaches. Our study revealed that NFE2L3 expression was significantly upregulated in multiple tumors and strongly associated with pathological stage and survival prognosis. NFE2L3 expression level of NFE2L3 had high diagnostic efficacy for the majority of tumors. Moreover, the methylation level of NFE2L3 was downregulated in most tumors and significantly associated with poor prognosis. We also analyzed genomic alterations of NFE2L3 in pan-cancer cells. NFE2L3 plays an essential role in immunomodulation in most tumors. In addition, we constructed a nomogram prognostic model for LIHC based on the expression of NFE2L3 and related clinical features, and the results showed that the model had high accuracy in predicting the OS of patients with LIHC. In summary, our study demonstrated the importance of NFE2L3 in the diagnosis and prognosis of pan-cancer, which could be conducive to further exploring its mechanism in tumorigenesis and development and provide a comprehensive analysis basis for cancer treatment in the future.

## Data Availability

The datasets presented in this study can be found in online repositories. The names of the repository/repositories and accession number(s) can be found in the article/Supplementary Material.

## Supplementary Material

Supplementary Figure S1 The correlation of NFE2L3 expression and disease-specific survival (DSS) in pan-cancer.

Supplementary Figure S2 The correlation between NFE2L3 expression and immunomodulators in pan-cancer **(A)** The correlation between NFE2L3 expression and immunoinhibitors in pan-cancer. **(B)** The correlation between NFE2L3 expression and immunostimulators in pan-cancer. **(C)** The correlation between NFE2L3 expression and MHC molecules in pan-cancer.

Supplementary Figure S3 Merged plots for GSEA analysis in multiple tumors. **(A)** BRCA; **(B)** CHOL; **(C)** ESCA; **(D)** HNSC; **(E)** KIRC; **(F)** PRAD; **(G)** UCEC; **(H)** THCA.

Supplementary Table S1 Baseline clinical data of TCGA-LIHC cohort.
