# Supplementary material for: Multi-Omics Analysis of Molecular Characteristics and Carcinogenic Effect of NFE2L3 in Pan-Cancer
Source: Front Genet. 2022 Jun 29;13:916973. doi: 10.3389/fgene.2022.916973 (PMC9284341; doi:10.3389/fgene.2022.916973)
Supplement: Supplementary file 1 [file Table1.DOCX]

| Characteristic | Low expression of NFE2L3 | High expression of NFE2L3 | p |
| --- | --- | --- | --- |
| n | 185 | 186 |  |
| T stage, n (%) |  |  | < 0.001 |
| T1 | 111 (30.2%) | 70 (19%) |  |
| T2 | 35 (9.5%) | 59 (16%) |  |
| T3 | 30 (8.2%) | 50 (13.6%) |  |
| T4 | 7 (1.9%) | 6 (1.6%) |  |
| N stage, n (%) |  |  | 1.000 |
| N0 | 125 (48.8%) | 127 (49.6%) |  |
| N1 | 2 (0.8%) | 2 (0.8%) |  |
| M stage, n (%) |  |  | 0.622 |
| M0 | 132 (48.9%) | 134 (49.6%) |  |
| M1 | 3 (1.1%) | 1 (0.4%) |  |
| Pathologic stage, n (%) |  |  | < 0.001 |
| Stage I | 105 (30.3%) | 66 (19%) |  |
| Stage II | 33 (9.5%) | 53 (15.3%) |  |
| Stage III | 32 (9.2%) | 53 (15.3%) |  |
| Stage IV | 4 (1.2%) | 1 (0.3%) |  |
| Tumor status, n (%) |  |  | 0.207 |
| Tumor free | 108 (30.7%) | 93 (26.4%) |  |
| With tumor | 70 (19.9%) | 81 (23%) |  |
| Gender, n (%) |  |  | 0.029 |
| Female | 50 (13.5%) | 71 (19.1%) |  |
| Male | 135 (36.4%) | 115 (31%) |  |
| Race, n (%) |  |  | 0.494 |
| Asian | 73 (20.3%) | 85 (23.7%) |  |
| Black or African American | 7 (1.9%) | 10 (2.8%) |  |
| White | 95 (26.5%) | 89 (24.8%) |  |
| Age, n (%) |  |  | 0.022 |
| <=60 | 77 (20.8%) | 100 (27%) |  |
| >60 | 108 (29.2%) | 85 (23%) |  |
| BMI, n (%) |  |  | 0.344 |
| <=25 | 85 (25.4%) | 92 (27.5%) |  |
| >25 | 85 (25.4%) | 73 (21.8%) |  |
| Residual tumor, n (%) |  |  | 0.902 |
| R0 | 165 (48.2%) | 159 (46.5%) |  |
| R1 | 8 (2.3%) | 9 (2.6%) |  |
| R2 | 1 (0.3%) | 0 (0%) |  |
| Histologic grade, n (%) |  |  | < 0.001 |
| G1 | 37 (10.1%) | 18 (4.9%) |  |
| G2 | 96 (26.2%) | 81 (22.1%) |  |
| G3 | 45 (12.3%) | 77 (21%) |  |
| G4 | 5 (1.4%) | 7 (1.9%) |  |
| Adjacent hepatic tissue inflammation, n (%) |  |  | 0.459 |
| None | 69 (29.5%) | 48 (20.5%) |  |
| Mild | 50 (21.4%) | 49 (20.9%) |  |
| Severe | 10 (4.3%) | 8 (3.4%) |  |
| AFP(ng/ml), n (%) |  |  | 0.014 |
| <=400 | 121 (43.5%) | 92 (33.1%) |  |
| >400 | 25 (9%) | 40 (14.4%) |  |
| Albumin(g/dl), n (%) |  |  | 0.393 |
| <3.5 | 41 (13.8%) | 28 (9.4%) |  |
| >=3.5 | 120 (40.4%) | 108 (36.4%) |  |
| Prothrombin time, n (%) |  |  | 0.428 |
| <=4 | 105 (35.7%) | 101 (34.4%) |  |
| >4 | 50 (17%) | 38 (12.9%) |  |
| Child-Pugh grade, n (%) |  |  | 0.895 |
| A | 124 (51.9%) | 93 (38.9%) |  |
| B | 13 (5.4%) | 8 (3.3%) |  |
| C | 1 (0.4%) | 0 (0%) |  |
| Fibrosis ishak score, n (%) |  |  | 0.151 |
| 0 | 47 (22.2%) | 27 (12.7%) |  |
| 1/2 | 14 (6.6%) | 17 (8%) |  |
| 3/4 | 12 (5.7%) | 16 (7.5%) |  |
| 5/6 | 46 (21.7%) | 33 (15.6%) |  |
| Vascular invasion, n (%) |  |  | 0.210 |
| No | 115 (36.5%) | 91 (28.9%) |  |
| Yes | 52 (16.5%) | 57 (18.1%) |  |
