# Supplementary figures and images for: Multi-Omics Analysis of Molecular Characteristics and Carcinogenic Effect of NFE2L3 in Pan-Cancer
Source: Front Genet. 2022 Jun 29;13:916973. doi: 10.3389/fgene.2022.916973 (PMC9284341; doi:10.3389/fgene.2022.916973)

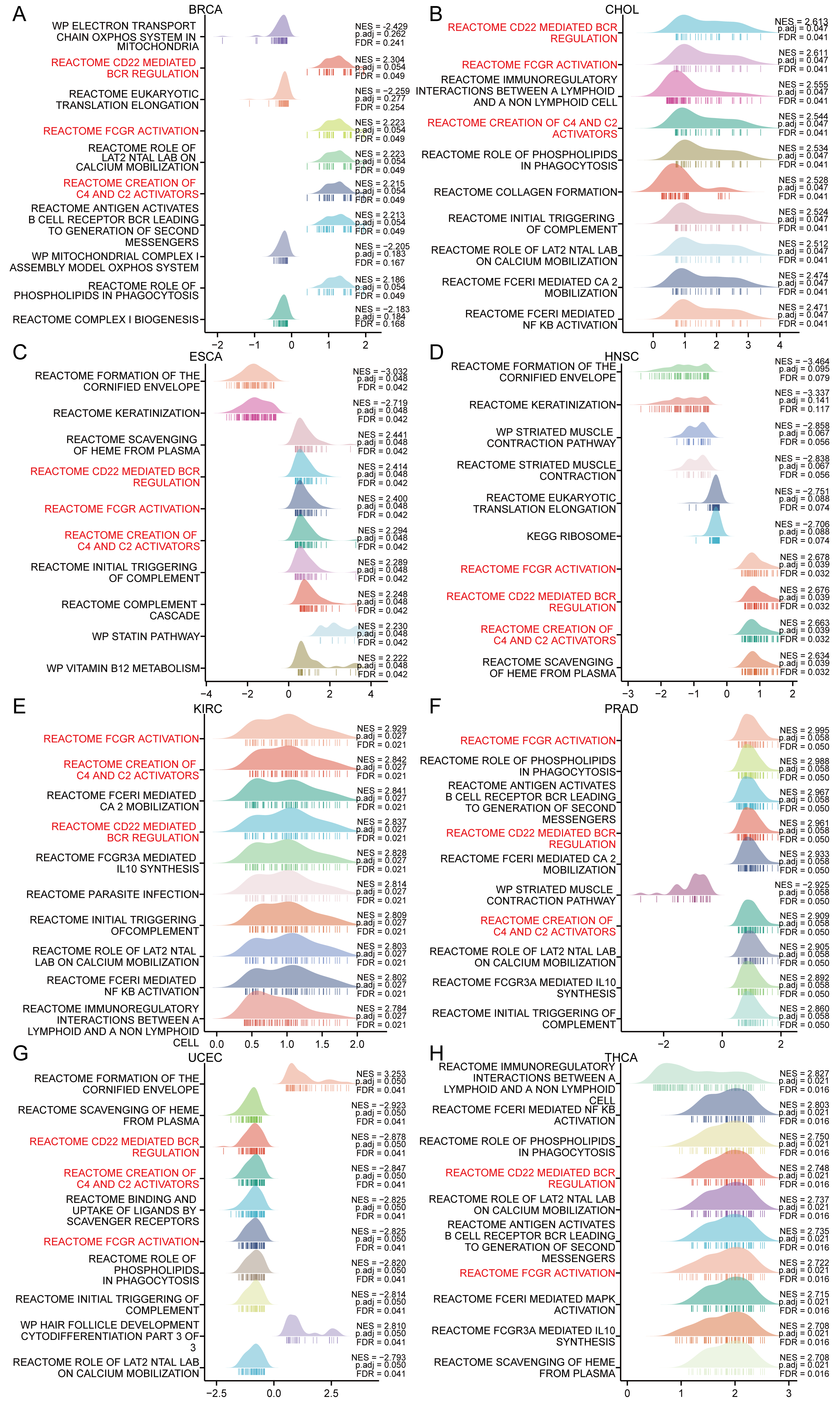

Supplement: Supplementary file 2 [file Image3.TIF]

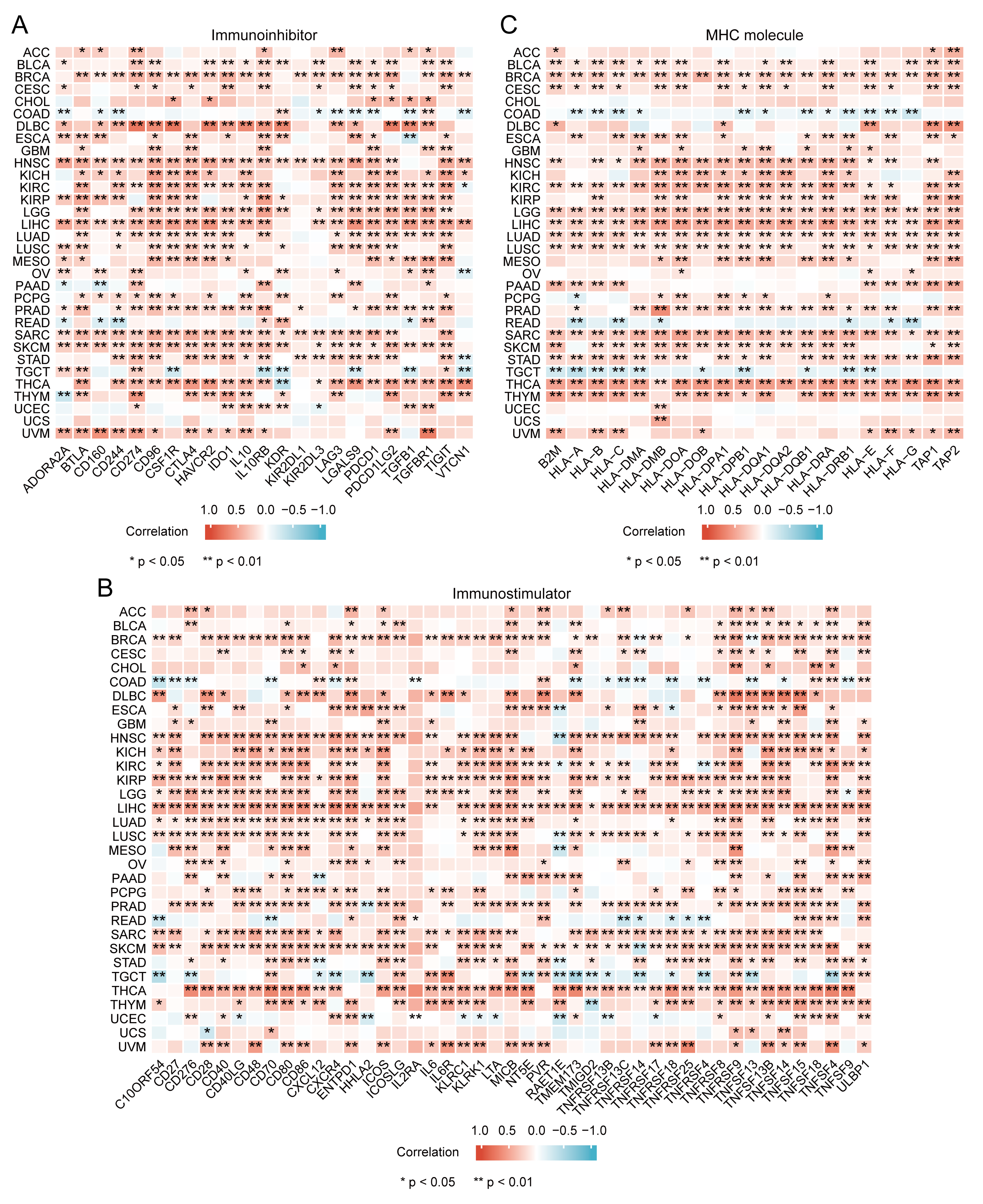

Supplement: Supplementary file 3 [file Image2.TIF]

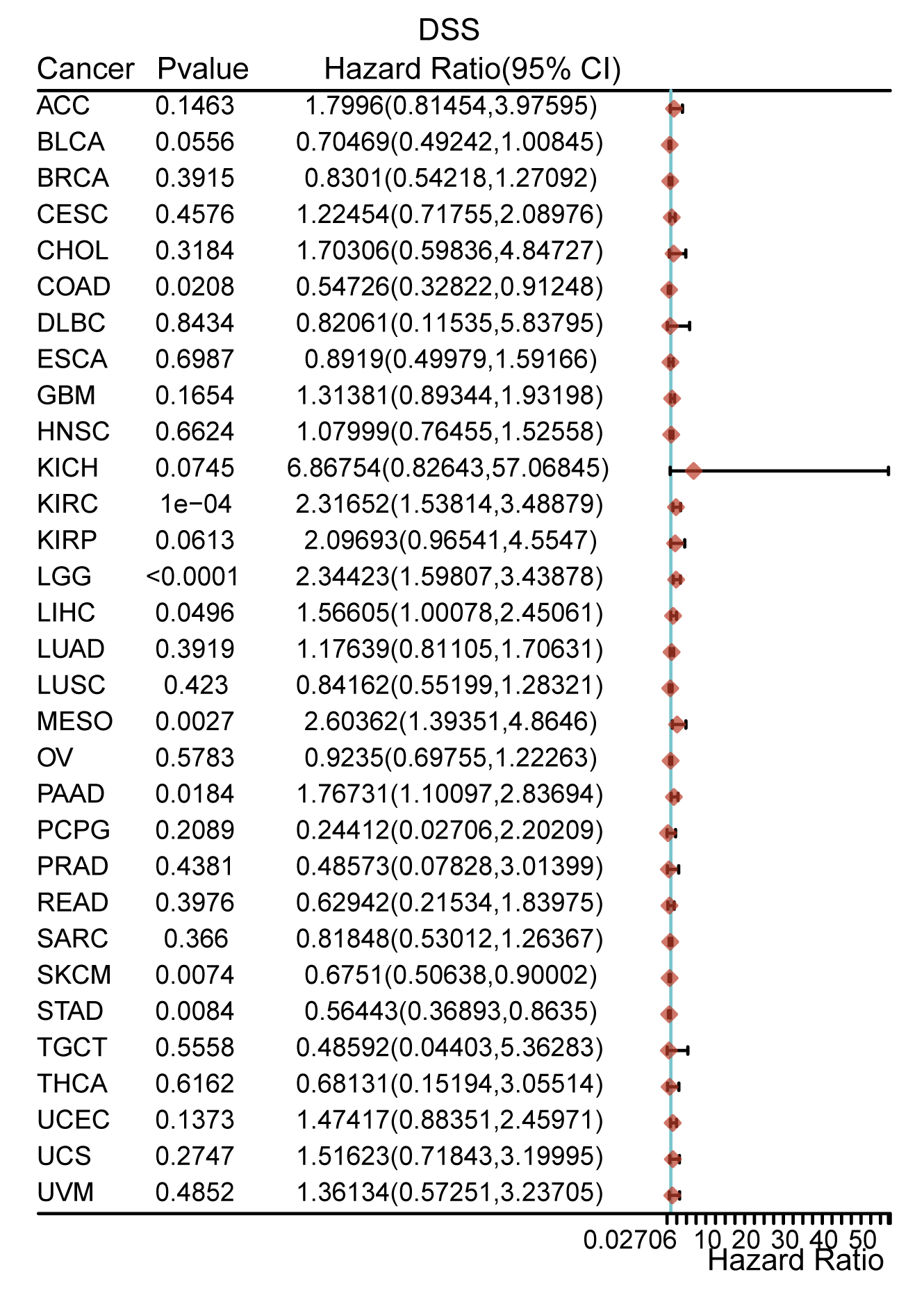

Supplement: Supplementary file 4 [file Image1.TIF]
